# The root of the East African cichlid radiations

**DOI:** 10.1186/1471-2148-9-186

**Published:** 2009-08-05

**Authors:** Julia Schwarzer, Bernhard Misof, Diethard Tautz, Ulrich K Schliewen

**Affiliations:** 1 Zoologisches Forschungsmuseum Alexander Koenig, Adenauerallee 160, 53113 Bonn, Germany; 2 Bavarian State Collection of Zoology, Münchhausenstr. 21, 81247 München, Germany; 3 Biozentrum Grindel & Zoologisches Museum, Martin-Luther-King-Platz 3, 20146 Hamburg, Germany; 4 Max-Planck-Institut für Evolutionsbiologie, August-Thienemann-Str. 2 24306 Plön, Germany

## Abstract

**Background:**

For decades cichlid fishes (Perciformes: Cichlidae) of the East African cichlid radiations (Teleostei: Cichlidae) have served as natural experimental subjects for the study of speciation processes and the search for potential speciation key factors. Despite numerous phylogenetic studies dealing with their intragroup relationships, surprisingly little is known about the phylogenetic placement and time of origin of this enigmatic group. We used multilocus DNA-sequence data from five nuclear and four mitochondrial genes and refined divergence time estimates to fill this knowledge gap.

**Results:**

In concordance with previous studies, the root of the East African cichlid radiations is nested within the so called "Tilapias", which is a paraphyletic assemblage. For the first time, we clarified tilapiine intragroup relationships and established three new monophyletic groups:"Oreochromini", "Boreotilapiini" and a group with a distribution center in East/Central Africa, the "Austrotilapiini". The latter is the founder lineage of the East African radiations and emerged at the Miocene/Oligocene boundary at about 14 to 26 mya.

**Conclusion:**

Our results provide the first resolved hypothesis for the phylogenetic placement of the megadiverse East African cichlid radiations as well as for the world's second most important aquaculture species, the Nile Tilapia, *Oreochromis niloticus*. Our analyses constitute not only a robust basis for African cichlid phylogenetics and systematics, but provide a valid and necessary framework for upcoming comparative phylogenomic studies in evolutionary biology and aquaculture.

## Background

African cichlid fishes (Perciformes: Cichlidae) constitute the most species rich vertebrate model system in evolutionary biology and ecology (reviewed in [1,2]). The spectacular radiations of the East African rift valley Lakes Malawi and Tanganyika, L. Victoria and surrounding smaller lakes and rivers, are best known for their exceptional diversity and efficient habitat and resource exploitation. [2]. Numerous studies on different aspects of speciation and the evolution of adaptive traits are based on East African cichlids, e.g. [3-6]. Identification of key factors [7] associated with the enormous evolutionary success of these radiations might improve our general understanding of speciation processes. For this a resolved phylogenetic framework is crucial [7]. Nevertheless, the closest relatives of the East African cichlid radiations (EAR) are still unknown, confusing interpretations of evolutionary trends in this group. This lack of knowledge can especially hinder comparative genomic studies and meta analyses, e.g. [1,5], which must rely on poorly resolved or poorly supported tree topologies. The monophyletic origin of African cichlids is supported by molecular and morphological analyses, as are five major monophyletic sublineages (Tylochromines, Hemichromines, Chromidotilapiines, Pelmatochromines, Haplotilapiines). A sixth lineage, the monotypic genus *Heterochromis*, is either regarded as a distant outgroup or as the sistertaxon to all remaining African cichlids [8-20]. It is further established that (1) the EAR, including the Nile Tilapia (*Oreochromis niloticus*), the world's second most important aquaculture species [21], are placed within the so called "Haplotilapiines" [18], of which internal relationships remain largely unresolved; and that (2) the root of the EAR is placed somewhere within a large subgroup of cichlid fishes, the so called "Tilapias" or "Tilapiines" [18,22]. Tilapiines are a widespread paraphyletic species assemblage including a few speciose and phenetically similar genera, i.e. *Tilapia*, *Oreochromis*, and *Sarotherodon*, as well as several less speciose and in some cases monotypic genera such as *Alcolapia*, *Tristramella*, *Danakilia*, *Iranocichla*, *Steatocranus*, *Gobiocichla *and *Chilochromis *[15,18,23]. Divergence time estimates for splits within the African cichlids are scarce and sometimes contradictory depending on the source of data. For example, fossil calibrated dating has resulted in much younger age estimates than Gondwana separation based dating (e.g. [24-26]). Reliable age estimates are not only required to link phylogenetic divergence with the palaeogeographical background but also to appraise the speed of evolutionary change associated with rapid speciation events. Until now age estimates for the origin of the East African radiations have been mainly based on geological information, e.g. on lake ages, assuming that divergence of endemic clades took place after the formation of lacustrine habitats [27,28]. Other estimates based on Gondwana fragmentation yield rather imprecise ages for terminal nodes [24,25] varying between 22 and 62 mya for the root of the EAR. The present study is designed to fill the gap between the rapidly increasing knowledge of various aspects shaping African cichlid evolution and the lack of a reliable phylogenetic background and divergence time estimates. In particular we intend to (i) establish a robust phylogeny for the paraphyletic group of Tilapias, (ii) identify the root of East African cichlid radiations, and finally (iii) estimate the root age of the primary East African radiation.

## Results

**Figure 1:**
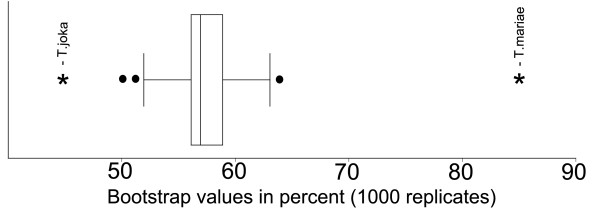
**Boxplot showing the results of the Homoplasy excess test**. The boxplot shows the distribution of bootstrap support values (%) for the Austrotilapiini. Each specimen was removed iteratively from the dataset (resulting in N = 63 experiments) and 1000 bootstrap replicates were calculated using ML. Outliers are shown as asterisks. Bootstrap support values clearly increased (from 56 initially to 86) after exclusion of *T. mariae*. This was the only that produced this effect.

The concatenated dataset included 56 taxa each with 6176 bp DNA sequence data derived from four mitochondrial and five nuclear loci (dataset A, additional file 1). Of these, 394 bp were excluded from the analyses due to alignment ambiguities in non-coding genes and saturation in the 3rd codon position of the mitochondrial ND2 locus, resulting in a final alignment of 5782 bp. A second dataset (B) was composed of 301 taxa and 993 bp of ND2 (additional file 2). The 3rd codon position was not excluded in this dataset, as taxon assignment to terminal groups rather than basal resolution was the focus. Parameters were estimated separately for each codon position. Dataset A had 1783 variable sites and empirical base frequencies of A = 0.269, C = 0.252, G = 0.228, T = 0.251. Dataset B had 707 variable sites and empirical base frequencies of A = 0.262, C = 0.357, G = 0.118, T = 0.262. The Bayes factor test [29] identified the HKY model as the best fitting model for all partitions except for nuclear exons (ENC1, Ptr, SH3PX3, Tmo4c4), which were assigned to GTR + Γ. As expected, nuclear genes gave a better resolution in the more basal splits whereas mitochondrial genes provided increased resolution in terminal groups. The leaf stability index revealed an unstable placement of *Tilapia mariae *(0.67 vs. 0.87 as next higher value) whereas all other taxa were comparatively highly supported. Exclusion of this taxon from further analyses increased the overall leaf stability index significantly (Wilcoxon matched pairs signed rank test, N = 62, z = -6.164, *p *< 0.001, leaf stability for all taxa > 0.90). Furthermore, exclusion of the ambiguous *T. mariae *yielded a clear increase of BPP and BS support values in affected clades. This effect was not evident during consecutive exclusion of all other taxa (Figure 1), thus *T. mariae *was excluded from all further analyses. Nevertheless, the topology of the remaining consensus trees in both ML and BI analyses remained unaffected.

### Phylogenetic relationships

**Figure 2:**
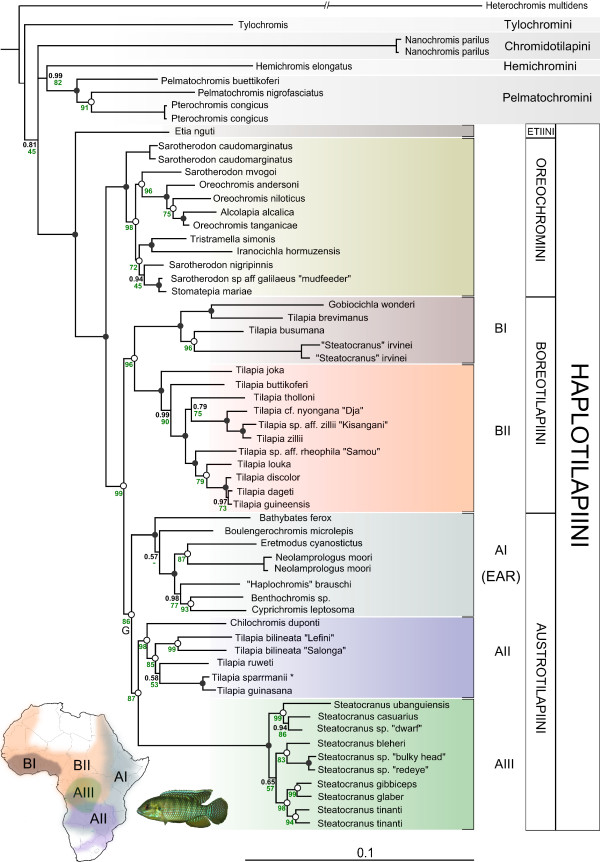
**Consensus BI Tree of the African cichlid phylogeny**. Consensus tree (50% majority rule) of the African cichlid phylogeny based on the concatenated dataset. The dataset comprises mitochondrial and nuclear sequences of nine independent markers. Green numbers at nodes refer to bootstrap-values (BS, 1000 replicates) of the ML run and black numbers to Bayesian posterior probabilities (BPP). Filled circles represent a 100% BS support and 1.00 BPP and empty circles 1.00 BPP and lower BS values. Major groups within the phylogeny were named based on either their center of geographic distribution (Austrotilapiini and Boreotilapiini) or based on taxonomic aspects (Oreochromini). The asterisk (*) in the tree marks the type species of the genus *Tilapia*. The leaf stability index exceeded 0.95 for all specimens, except for clade AI (all taxa 0.90). Note that for clade AI only representatives of the EAR are included. The results presented here were verified using a more detailed taxon sampling based on ND2 (see additional file 3). The map in the lower left corner shows major distribution ranges for Austro- and Boreotilapiini. Pictured is *T. ruweti*.

Trees obtained from ML and BI analyses were highly congruent and nodes were supported for all major clades. Both approaches corroborated the monophyly of the Haplotilapiini (100/1.00) whereas sister group relationship of this group within the African cichlids gained low BS and BPP values (45/0.74, Figure 2). Within the "Haplotilapiini the following topology was highly supported (BS and BPP = 99): (a) *Etia nguti *was sister group to all other Haplotilapiines. (b) The mouthbrooding genera *Oreochromis*, *Sarotherodon*, *Iranocichla *and *Tristramella *formed a monophyletic group, hereafter named "Oreochromini", after the most species rich genus within this group *Oreochromis*. The Oreochromini were sister to the substrate-brooders (clades BI, BII, AII and AIII) as well as to clade AI, comprising substrate and mouthbrooding representatives of the East African radiations (Figure 2). (c) A clade comprising clade AI (100/1.00), *Tilapia sensu stricto *(AII, 98/1.00), and *Steatocranus *from the Congo Basin (AIII, 100/1.00) formed the sister group to remaining Haplotilapiines distributed mainly in the East/Central/Southern part of Africa. In recognition of its distribution, this group is called "Austrotilapiini", in contrast to the "Boreotilapiini" with a predominantly West/Central African distribution (Figure 2). Within the Boreotilapiini, a clade consisting of *Gobiocichla wonderi*, *Tilapia brevimanus*, *Tilapia busumana *and *"Steatocranus" irvinei *(BI, 100/1.00) and a clade comprising the *Tilapia (Coptodon) *subgenus (sensu [30]) as well as *T. joka *and *T. buttikoferi *(BII, 100/1.00, Figure 2) appeared monophyletic and emerged as well supported sister groups (96/1.00). Within the Austrotilapiini sister group relationships were consistent and moderately well supported (86/1.00 and 87/1.00 respectively). All major clades were confirmed as monophyletic in a larger phylogenetic framework based on ND2 (additional file 3).

### The phylogenetic placement of the East African radiations

Clade AI, comprising the EAR, appeared as sister group to the remaining Austrotilapiini (Figure 2). The mitochondrial dataset supported a sister group relationship between AI and the Congolian genus *Steatocranus *(AIII), though with low support values (63/0.80), whereas the nuclear dataset in accordance with the concatenated dataset, favored the above mentioned relationship ((69/0.95) and (87/1.00) respectively). Discordant phylogenetic signal was evident in 6% and 7% of the bootstrap replicates, favoring either a placement as sister to monophyletic Boreotilapiini and Austrotilapiini (6%) or a sister group relationship to Boreotilapiini alone (7%). All remaining hypotheses were supported with less than 1% (additional file 4). The 6% signal was only detectable in the nuclear non-coding intron S7: without this marker the signal was hardly detectable (additional file 5). No conflicting signal was detectable in 2000 randomly chosen BI topologies.

### Divergence time estimates

**Figure 3:**
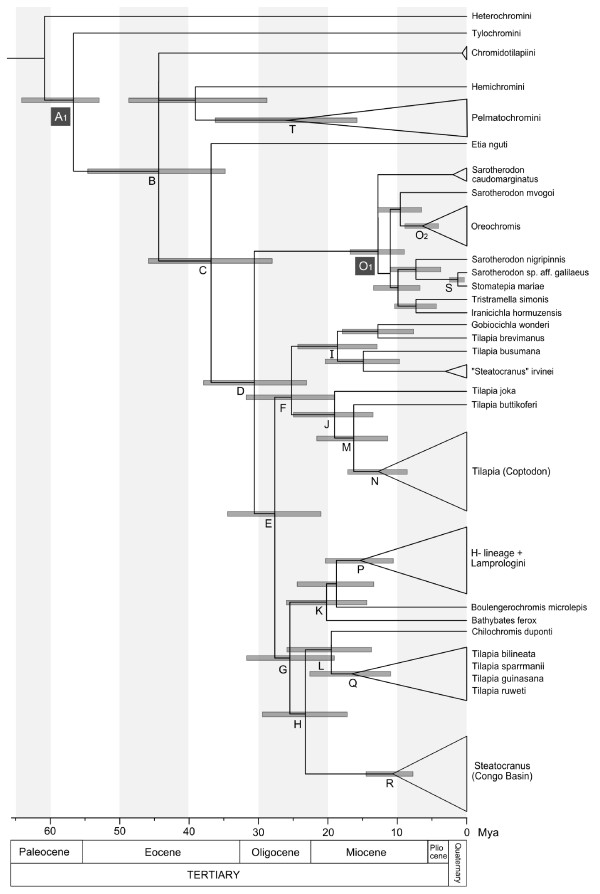
**Chronogram showing divergence time estimates**. The chronogram was calculated based on the BI consensus tree. Divergence times were estimated using a partitioned Bayesian analysis implemented in BEAST. The following time constraints were used: A_1 _53–84 mya (uniform prior), published age estimate based on non-cichlid fossils [24] and O_1 _5.98 mya (lower bound), the age estimate for *Oreochromis lorenzoi*† [31]. The chronogram shows 95% credibility intervals (HPC, grey bars). For nodes marked with letters, age estimates (95% HPC and mean heights) are given in Table 1. Calibration points (O_1 _and A_1_) are marked with black squares. For simplification clear monophyletic groups were combined (shown as triangles).

Divergence time estimates yielded broadly consistent results (Table 1). Preliminary analyses indicated a younger age for node A (Figure 3) than represented by prior A_2 _(71–89 mya, Gondwana calibration from [24]) and the age estimates for most recent ancestor of *Oreochromis *(Node O_2_, Figure 3) were younger (minimum age 4.18 mya, Table 1) than the age of the *Oreochromis lorenzoi*† fossil [31]. Thus, final analyses were performed using priors O_1 _(lower bound 5.98 mya at the base of all Oreochromini, Figure 3) and A_1 _(53–84 mya, teleost fossil calibration from Azuma et al. [24]). The mean standard deviation width of the 95% highest posterior density (HPD) was 12.07–5.32 mya and the precision of the estimate was highly correlated with node age (Pearson correlation, *p *< 0.001, r = 0.703, N = 21), pointing to more precise younger ages. The age of the most recent common ancestor of the Haplotilapiini was estimated at about 37 (28–46) mya (Figure 3, node C). Mean ages for the three major clades within the Haplotilapiini were estimated at about 25 (19–32) mya for both Austrotilapiini and Boreotilapiini (nodes F and G) and 13 (9–17) mya for the constrained Oreochromini (node O_1_, Figure 3). The age for the East African radiations, including the ancient lineages Bathybatini and *Boulengerochromis *was estimated at 20 (14–26) mya (node K) and the subclade comprising the H-lineage and "Lamprologini" was estimated to have emerged at 15 (11–20) mya (node P). In a second analysis Gondwana estimates, following [24], were included for calibration point A (A_3_: 53–89 mya, Table 1). Results were highly congruent with the first run using fossil calibrations even though confidence intervals increased. The alternative algorithm based on penalized likelihood revealed highly congruent results with those obtained by the Bayesian approach (Table 1).

**Table 1:** Date estimates resulting from different molecular clock approaches

**Date estimates in Myr**								
**Node**^§^	**Bayesian Inference (BEAST)**						**Penalized likelihood**	

	**This study**^1^		**This study**^2^		**Genner et al**. [25]	**Gondwana**	**This study**^1^	**This study**^2^
**A**_1_	**56.7**	(53.0, 64.2)	**66.5**	(53.0, 85.2)	63.7 (node N)	(46.6, 79.6)	53.0	53.4
B	**44.4**	(34.8, 54.6)	**55.7**	(40.9, 74.5)			48.8	49.2
C	**36.8**	(28.0, 45.9)	**46.9**	(32.9, 63.2)			37.1	37.4
D	**30.6**	(23.1, 37.9)	**39.6**	(27.9, 54.0)	46.4 (node M)	(31.9, 61.7)	28.4	28.6
E	**27.6**	(21.0, 34.5)	**35.8**	(24.9, 48.9)			24.6	24.8
F	**25.3**	(18.9,31.8)	**32.8**	(22.4, 44.9)			22.6	22.8
G	**25.5**	(19.0, 31.7)	**33.0**	(22.6, 45.0)			23.1	23.2
H	**23.3**	(17.2, 29.5)	**30.1**	(20.3, 41.3)			21.9	22.1
I	**18.6**	(12.9, 24.3)	**24.3**	(15.8, 34.2)			15.6	15.7
J	**19.0**	(13.5, 24.9)	**24.7**	(15.9, 34.8)			16.4	16.7
K	**20.2**	(14.4, 26.0)	**26.1**	(17.5, 36.7)	35.6 (node L)	(22.3, 50.6)	18.1	18.2
L	**19.5**	(13.7, 25.9)	**25.3**	(16.5, 36.3)			19.7	19.9
M	**16.3**	(11.3, 21.6)	**21.3**	(13.5, 30.3)			14.2	14.4
N	**12.8**	(8.6, 17.1)	**16.7**	(10.3, 23.9)			11.2	11.3
**O**_1_	**12.8**	(8.9, 16.8)	**21.4**	(12.9, 31.1)			19.4	19.5
**O**_2_	**6.4**	(4.1, 8.9)	**9.7**	(5.5, 14.4)			8.4	8.5
P	**15.4**	(10.6, 20.4)	**20.0**	(12.8, 28.4)	29.5 (node K)	(17.7, 43.2)	14.3	14.3
Q	**16.6**	(10.9, 22.7)	**21.7**	(13.1, 31.4)			17.5	17.6
R	**10.7**	(7.4, 14.1)	**14.4**	(9.0, 20.6)			5.7	5.7
S	**1.31**	(0.4, 2.5)	**1.8**	(0.4, 3.4)			2.6	2.6
T	**25.5**	(15.8, 36.2)	**32.3**	(17.5, 48.4)			35.3	35.6

Single dating points (mean height) and confidence intervals (95%HPC) are shown for runs with (1) and without (2) the cichlid fossil calibration point. Prior A was constrained either with 53 to 84 mya (A_1_, run 1) or with 53–89 mya (A_3_run2). **§ **Letters correspond to node labels in Figure 3.

## Discussion

With this well-supported phylogeny and consistent divergence time estimates for the ancestors of the most diverse group of African cichlids a stable foundation is laid for further studies on this prime model system in evolutionary biology. Our results clearly show that the genus *Tilapia *is paraphyletic, and that previously proposed tilapiine subgenera, summarized in [32], need revision. As this is beyond the scope of the present study, we propose in accordance with good practice in cichlid taxonomy to use the genus name *Tilapia *Smith, 1840 only for *Tilapia *sensu stricto, i. e. the small ingroup of southeastern species containing the type species *Tilapia sparrmanii*, along with *T. ruweti*, *T. baloni*, and *T. guinasana*. Pending a thorough revision all other members should be referred to as "Tilapia" (in quotation marks). The informal designation of identified clades Etiini, Oreochromini, Austrotilapiini and Boreotilapiiini will facilitate discussion of haplotilapiine monophlyetic groups in the absence of a full taxonomic revision and renders the previously used term "Tilapiini" meaningless in the phylogenetic context. A list of all currently valid tilapiine species level taxa and their placement with respect to the newly named clades is provided (additional file 6) and will be available in a regularly updated version under .

### Phylogenetic relationships of African cichlids

Resolving relationships of African cichlids has always been challenging. While phylogenetic relationships between and within the African Great Lake radiations [1,2] and riverine Haplochromines [27,33,34] are comparatively well understood, little was known about the broader phylogenetic framework for the most speciose group of cichlids [1,22]. Most often the so called Tilapiines were discussed as precursors of the East African cichlid radiations [1,22]. Several morphological studies classified different tilapiine genera into various numbers of subgenera largely based on overall similarity of character states rather than on unambiguous apomorphies [23,32]. However, the diversity of this heterogeneous group was comparatively poorly represented in molecular phylogenetic studies [8], but see [15,17,35]. A recent work based on the mitochondrial ND2 marker [15] accentuated the paraphyletic origin of the genera *Tilapia *and *Sarotherodon*, but did not recover well-supported deeper phylogenetic relationships. We present the first largely resolved phylogeny of African cichlids with emphasis on tilapiine cichlids including 47 ingroup and 7 outgroup species (Figure 2). Phylogenetic analyses revealed congruent and largely well-resolved topologies supporting a monophyletic origin of the Haplotilapiini, comprised of all tilapiine cichlids as well as the East African radiations. Relationships of Haplotilapiines to basal African cichlid tribes were only weakly supported, possibly due to high genetic distances compounding homoplastic signal amongst the most ancient nodes. In accordance to previous results the sister group to all remaining Haplotilapiines was the monotypic taxon *Etia nguti *from the Cross River in Cameroon [18]. Earliest divergence within the Haplotilapiini separates the mouthbrooding, almost pan-African Oreochromini from predominantly substrate brooding tilapiines and the EAR. The latter formed two monophyletic clades with largely non-overlapping distribution, one with a center in West/Central Africa (Boreotilapiini), and one in East/Central Africa (Austrotilapiini, Figure 2). Remarkably a comparable distribution pattern is evident in cyprinodont killifish [36,37], explained by a marine incursion in the late Palaeocene at about 92-52 mya [38,39] separating West Africa from the East and Central part. However, estimates for the Haplotilapiine clades are substantially younger, with 28 (21–35) mya (Figure 3, node E) for the separation of Boreo- and Austrotilapiini and a subsequent diversification at 25 (19–32) mya (Nodes F and G). This even holds true without a fossil prior (Table 1). The estimated ages are concordant with the East African aridification at 33-20 mya [40,41], which influenced distribution patterns of the African fauna and flora, i.e. rainforest trees [42] and caecilian amphibians [40]. However, the influence of the drought on freshwater systems possibly inhabited by the ancestors of the Austro- and Boreotilapiini is not known at this time, leaving room for speculation about the evolution of this distribution pattern. The position of *Tilapia mariae *remained ambiguous in our analyses, which is reflected by a low leaf stability index. The predominant phylogenetic signal resulted in its placement as sistertaxon to Boreotilapiines, but depending on the algorithm used, it was also sometimes resolved as sister to Austrotilapiines (additional file 4). A possible explanation for this could be an ancient hybrid origin of *T. mariae*, causing discordant phylogenetic signals in our dataset. Indeed, the distribution of the clade represented by *T. mariae *and its sistertaxon *T. cabrae *is intermediate between Austrotilapiines and Boreotilapiines (Figure 2). A more detailed analysis is necessary to elucidate this pattern.

### The origin of the East African radiation

The root of the East African radiations (EAR) within the substrate brooding tilapiine cichlids (Figure 2) is corroborated with high support values. These results are consistent with earlier analyses based on limited taxon sampling or fewer loci, e.g. [8,22], obtaining a closer sister group relationship of *Tilapia*/*Steatocranus *to the EAR than the mouthbrooding Oreochromini. Whereas the *Steatocranus *radiation of the Congo Basin forms a monophyletic clade, the genus *Tilapia *is clearly paraphyletic [15]. *Tilapia *taxa included in the study of Terai et al. [22] have affinities with the more distantly related Boreotilapiini in our analyses. A closer phylogenetic relationship of the EAR to the (Austrotilapiini), comprising the Congolese *Steatocranus *and a clade composed of *Tilapia s.str*., *T. bilineata *and *Chilochromis*, is corroborated (Figure 2). Biogeographically this is plausible, because Austrotilapiini and the EAR largely overlap in their distribution around the East African Lakes. In this area, the only representative of the Boreotilapiini present is *T. (Coptodon) rendalli*. The divergence of the EAR clade was estimated at 20 (14–26) mya (Figure 3, Node K) including the ancient lineages and at 15 (11–20) mya (Figure 3, node P) for the more derived lacustrine and riverine radiations. Though only slightly overlapping, the latter age estimate would be congruent with an origin of the derived lineages in an emerging Lake Tanganyika, estimated at 9–12 mya [43]. Alternative age estimates using Gondwana fragmentation calibrations or an alternative dating algorithm (penalized likelihood) point to an older age for this node at 20 (13–28) mya and 14 mya, respectively (Table 1). These estimates favor the alternative hypothesis of an origin of derived lineages prior to the formation of Lake Tanganyika, in surrounding rivers or peripheral palaeolakes and subsequent independent colonization [25]. Possibly, an increased taxon sampling with a multi-locus dataset would render more precise age estimates and remove this remaining uncertainty.

## Conclusion

Here, we provide the first reliable phylogenetic placement of one of the most important model organisms in evolutionary biology, the East African cichlids. We show that they are sister group to geographically proximate tilapiine cichlids with a main distribution center in East/Central Africa and that the whole group emerged in the late Oligocene/early Miocene. The dataset provided here constitutes not only a stable basis for critical testing of divergence dates for basal EAR lineages from their tilapiine precursors [44] but also a critical template for future phylogenomic and comparative studies based on African cichlids.

## Methods

### Samples and Sequences

A total of 63 specimens of 54 species were included, representing all major groups of African cichlids, focusing on Haplotilapiines sensu Schliewen & Stiassny [18]. To serve as nested outgroups, members of all basal African lineages (Tylochromini, Chromidotilapiini, Pelmatochromini, Hemichromini) were added. As several recent molecular and morphological studies support the basal position of *Heterochromis multidens *with respect to the rest of the African cichlid radiation [10,16,45], this taxon served as outgroup. Total genomic DNA was isolated from fin clips or muscle tissue using the Qiagen Tissue Extraction Kit (DNeasy Tissue Extraction Kit) following the manufacturer's protocol. The following mitochondrial markers were amplified and sequenced: partial mitochondrial 12S and 16S genes and the intervening sequence between them, and ND2. Additionally, four nuclear protein coding genes (ENC1, Ptr, SH3PX3 and Tmo4c4) and the first intron of the ribosomal protein coding gene S7 were amplified and sequenced. The programs BioEdit (ClustalW) and MUSCLE v.3.6 were used for sequence alignment, followed by a control for ambiguous alignment positions using ALISCORE v.0.2 under default settings [46]. ALISCORE checks for random sequence similarity using MCMC and a sliding windows approach. Under this regime, similarity profiles based on pairwise comparisons of sequences were calculated. Ambiguous positions were summarized in a consensus profile along the alignment [46] and subsequently removed from all analyses.

Coding genes were translated into amino acid sequences to check for stop-codons or frame shifts and datasets were checked separately for saturation at each codon position. Base frequencies were equal for all markers (Chi-square tests, df = 183, all *p *> 0.9). The combined dataset of all sequenced markers resulted in a data matrix of 6176 total bp comprised of 12SrRNA: 349 bp, 16SrRNA:543 bp, 12S/16S:1245 bp, ND2: 1014 bp, ENC1: 725 bp, Ptr: 691 bp, SH3PX3: 681 bp, Tmo4c4: 425 bp and S7 (first intron): 503 bp. In addition, a second dataset of 263 ND2 sequences (900 bp) retrieved from Genbank and 38 newly sequenced ND2 sequences was generated (additional file 2), resulting in high coverage over all major African cichlid tribes, some of which are not present in data set A. ND2 was chosen because this marker was available on GenBank for a representative sampling of African cichlids. The third codon position was saturated between in- and outgroups in dataset B, but because the focus of this analysis was the identification of terminal clades (younger splits), third positions were not excluded. Data were partitioned according to 1st, 2nd and 3rd codon position and all parameters were estimated separately. A ML phylogeny was constructed with RAXML v.7.0.3 using the rapid hill climbing bootstrap algorithm with 1000 replicates and following ML search. Branches not supported by 50% bootstrap values were collapsed.

### Phylogenetic reconstruction

Bayesian Inference (BI) and Maximum Likelihood (ML) approaches were used for phylogenetic inferences. The combined dataset was partitioned according to coding vs. non-coding and mitochondrial vs. nuclear genes yielding four partitions, i.e. two partitions for mitochondrial genes (rRNA and 1st and 2nd codon position of ND2) and two for nuclear genes (Exons and Intron). The third codon-position of ND2 was excluded from phylogenetic analyses (dataset A), as previous tests showed saturation between Haplotilapiine and basal taxa (data not shown). For each partition model parameters were estimated separately. For BI, best fitting models of sequence evolution were estimated using the Bayes Factor Test [29]. Bayesian analyses were performed using MrBayes v.3.1.2 [47] with eight parallel runs each over 10^6 ^generations starting with random trees and sampling of trees every 1000 generations. To ensure convergence the first 10^5 ^generations of each run were treated as burn-in and excluded. The remaining trees from all Bayesian analyses were used to build a 50% majority rule consensus tree. The GTR + 3 model, implemented in the program RAxML v.7.0.3 [48] was used for Maximum Likelihood analyses. Branch support was evaluated for the best scoring ML tree using non-parametric bootstrapping (BS) consisting of 1000 pseudoreplicates (using RAxML) and Bayesian posterior probabilities (BPP).

### Testing alternative phylogenetic hypotheses

To test for unreliably placed taxa the leaf stability index [49] was calculated for all taxa based on 1000 bootstrap trees using the program Phyutility v.2.2. [50]. This index is a good measure of the consistency of a taxon's position relative to other taxa across bootstrap replicates. Using the same program, branch attachment frequencies were calculated for clades with low support values (*BS *< 90) using 1000 bootstrap trees and the ML topology as well as the first 2000 BI topologies (after burn-in) and the BI topology. Following Seehausen [51], we applied a tree-based method to test for excess homoplasy in our dataset, possibly introduced by taxa of ancient hybrid origin. The inclusion of a hybrid taxon would be expected to increase internal conflict in the tree and diminish support values for affected nodes owing the reticulate nature of the process [51]. To test for this possibility, each taxon was successively removed from the dataset (N = 63 experiments) and subsequently a likelihood run (using RAxML) under the GTR + 3 model with 1000 rapid bootstrap replicates was conducted for each resulting dataset. The resulting trees and bootstrap support values for the focus clades were checked manually.

### Divergence time estimates

Date estimates were calibrated using two age constraints. One calibration point (O) was based on the fossil record of *Oreochromis lorenzoi*† [31] from the Early Miocene of the Baid Formation (5.98 mya, [52]). The second calibration point, assigned to the split between *Tylochromis *and the remaining African cichlids (except *Heterochromis multidens*), corresponds to the 95% credible interval estimates for African Cichlidae from Azuma et al. [24], (exact dates were kindly provided by Y. Kumazawa and Y. Azuma, pers. comm.). Estimates based on both non-cichlid teleostean fossils (A_1 _53–84 mya) and Gondwana fragmentation (A_2 _71–89 mya) were taken. An exponential prior using a zero offset of 5.58 mya (marking the minimum age) with a mean of 1 was used for the fossil calibration point and a uniform prior with upper and lower bounds either from 53 to 84 mya (A_1_), 71 to 89 mya (A_2_) or a combination of both with 53 to 89 mya (A_3_) [24] were fixed prior to analyses. As the distinction between *Oreochromis *and *Sarotherodon *is based on characters that are often not preserved in fossils [23], at least two possible placements for the *Oreochromis lorenzoi*† fossil in the phylogenetic framework exist. Most conservative is a placement at the base of all mouthbrooding Tilapiines (O_1_) or, less so is a placement at the base of the genus *Oreochromis *(point O_2_). *Oreochromis lorenzoi*† [31] is in our point of view one of the few reliable cichlid fossils suitable for calibration, as the type specimens are in a well preserved state and all key traits necessary for identification are recognizable. Its phylogenetic placement within the African cichlid phylogeny is less ambiguous than for other fossils, as the Oreochromini are a clearly monophyletic group (Figure 2). Unfortunately this is not the case for most other African cichlid fossils, which often lack diagnostic characters necessary for a precise assignment to cichlid tribes (for a more detailed discussion see additional file 7). Divergence time analyses were conducted using a log-normal distributed relaxed molecular clock MCMC approach [53] as implemented in BEAST v.1.4.8 [54]. For all calculations data were partitioned as described earlier and the BI topology was used as starting tree. Separate substitution models were used for each partition based on the results of the Bayes Factor test. A pure birth model (Yule) was assigned as prior for the branching process and two independent and identical runs were conducted for each BEAST setup for 30^6 ^generations. Convergence of parameters was checked using Tracer v.1.4 [55]. The first 10% of generations were discarded as burn-in and the effective sample size (ESS) was checked for good mixing of the MCMC. All exceeded 200 for all model parameters. Divergence dates were also estimated using penalized likelihood [56] as implemented in the program r8s v.7.1 [57]. The optimal smoothing parameter was 63 for each run determined by a cross-validation approach [56]. All runs were conducted several times with different sets of constraints to evaluate the influence of different calibration points. As expected inclusion of the fossil calibration point produced slightly younger but also narrower confidence intervals for all ages (additional file 8 and Table 1). Two alternative placements of *Oreochromis lorenzoi*† within the topology resulted in slightly different age estimates, with younger ages when the calibration point was set at the root of all Oreochromini. Using the penalized likelihood approach no difference in age estimates was observed for different placements of *Oreochromis lorenzoi*†. Overall, age estimates largely overlap independent of the priors used (additional file 8).

## Authors' contributions

UKS and JS designed the study. JS carried out the molecular work. JS, BM and UKS designed and conducted the analyses. All authors contributed to the preparation of the manuscript. They read and approved the final version.

## Supplementary Material

Additional file 1 **Taxa list and GenBank accession numbers for dataset A**. List of all taxa and genes (with GB accession numbers) included in dataset A. Click here for file

Additional file 2 **Taxa list and GenBank accession numbers for dataset B**. List of all taxa and accession numbers for ND2 included in dataset B. **§ **Letters correspond to node labels (if shown) in phylogenetic tree in additional file 3. Click here for file

Additional file 3 **Maximum likelihood Phylogeny based on dataset B**. Maximum likelihood phylogeny for dataset B based on 992 bp of ND2. Sequences were taken from GenBank (N = 263) and additional taxa from dataset A (N = 38) were also included. Focus clades are marked with black bars and BS support values are given only for those clades. All focus clades (well supported clades from dataset A) were recovered as monophyletic in this tree, despite lower data density and higher taxon sampling. One sequence of *Tilapia discolor *taken from GenBank is nested within *T. busumana *in clade BI instead of being sister to our conspecific and positively identified *T. discolor*. As no vouchers are available for this tissue, we assume that this discrepancy is a result of either misidentification or mitochondrial introgression of sympatric *T. busumana *is the reason for this discrepancy. Click here for file

Additional file 4 **Branch attachment frequencies in bootstrap replicates**. Alternative positions of the single unstable taxon, *T. mariae *(a), and the EAR (b) in 1000 bootstrap topologies. The numbers, plotted on the ML tree, indicate fractions of bootstrap trees in which alternative branching patterns occur. Click here for file

Additional file 5 **Results of the Approximately unbiased test**. Results of the approximately unbiased (AU) test for alternative phylogenetic placements of the EAR with and without the nuclear intron S7. The topologies tested were taken from the branch attachment frequency test (topology 1–5) or were consensus topologies based on solely mitochondrial or nuclear markers (topology 6–7). Additionally, a topology with a polytomy at the base of the Austrotilapiini was tested (topology 8). Click here for file

Additional file 6 **Informal classification of African Cichlid fishes**. Informal classification of African cichlid fishes. Click here for file

Additional file 7 **Supplementary Information**. Supplementary information for lab work (amplification, purification and sequencing of PCR products) and the choice of calibration priors for divergence time estimation. Click here for file

Additional file 8 **Prior influence on divergence time estimates**. The effects of different age constraints on the estimation of divergence times using BEAST. Bars indicate age ranges (95% credibility intervals) of different BEAST runs using either one single prior on the root (A_3_: 53–89 mya, based on published time intervals from [24]) or two priors, including the *Oreochromis lorenzoi *fossil (lower bound 5.98 mya) at two possible positions (O_1 _and O_2_) in the phylogeny (Figure 2). Using solely the root prior increases credibility intervals and renders the whole age estimation older. Inclusion of the fossil prior shifts intervals to a younger age. Large overlaps in estimates unite all three results and increase the plausibility of the presented results. Alternative positions of the *Oreochromis lorenzoi*† prior had no effect in age estimates using penalized likelihood (r8s). Click here for file
