# Supplementary figures and images for: The root of the East African cichlid radiations
Source: BMC Evol Biol. 2009 Aug 5;9:186. doi: 10.1186/1471-2148-9-186 (PMC2739198; doi:10.1186/1471-2148-9-186)

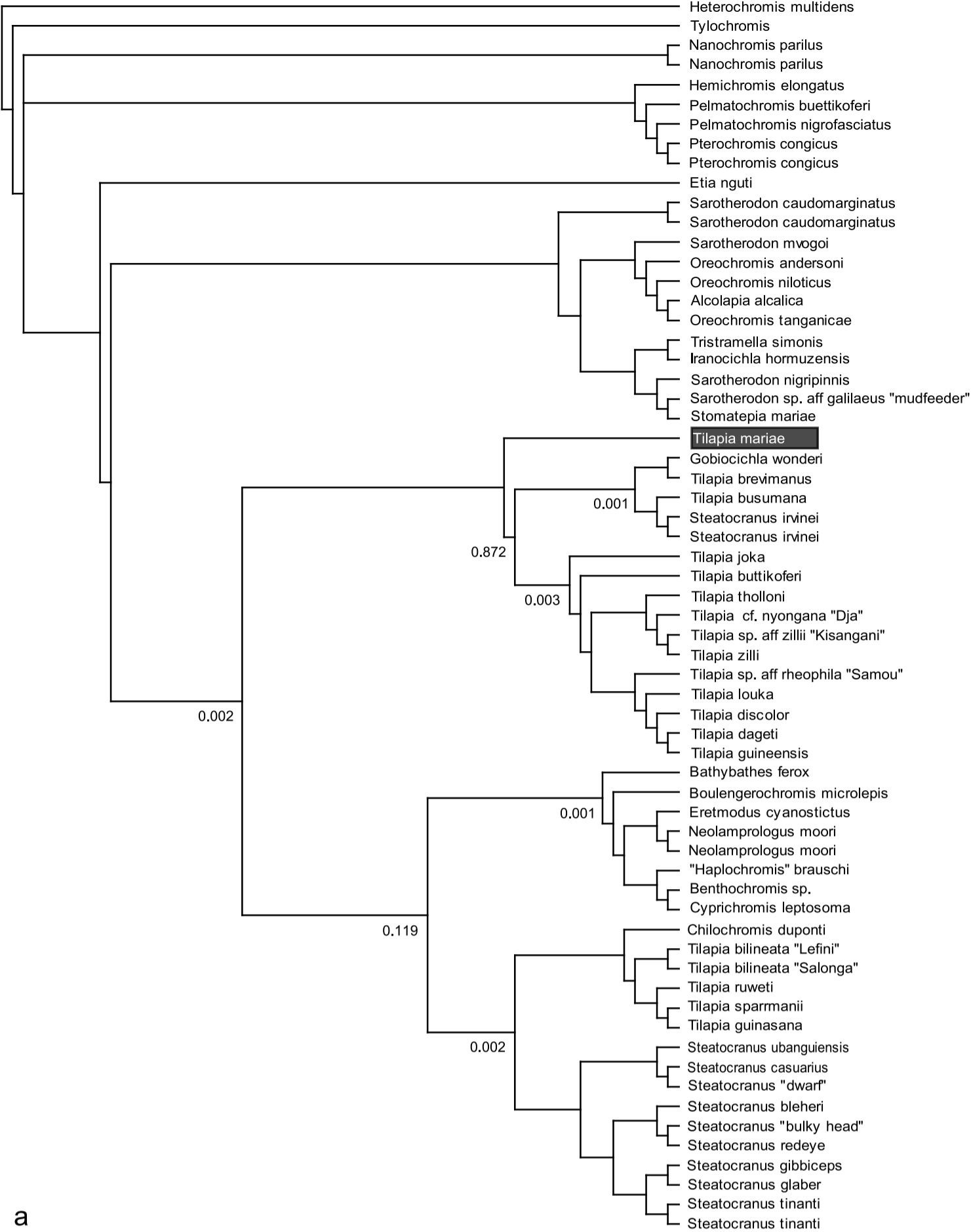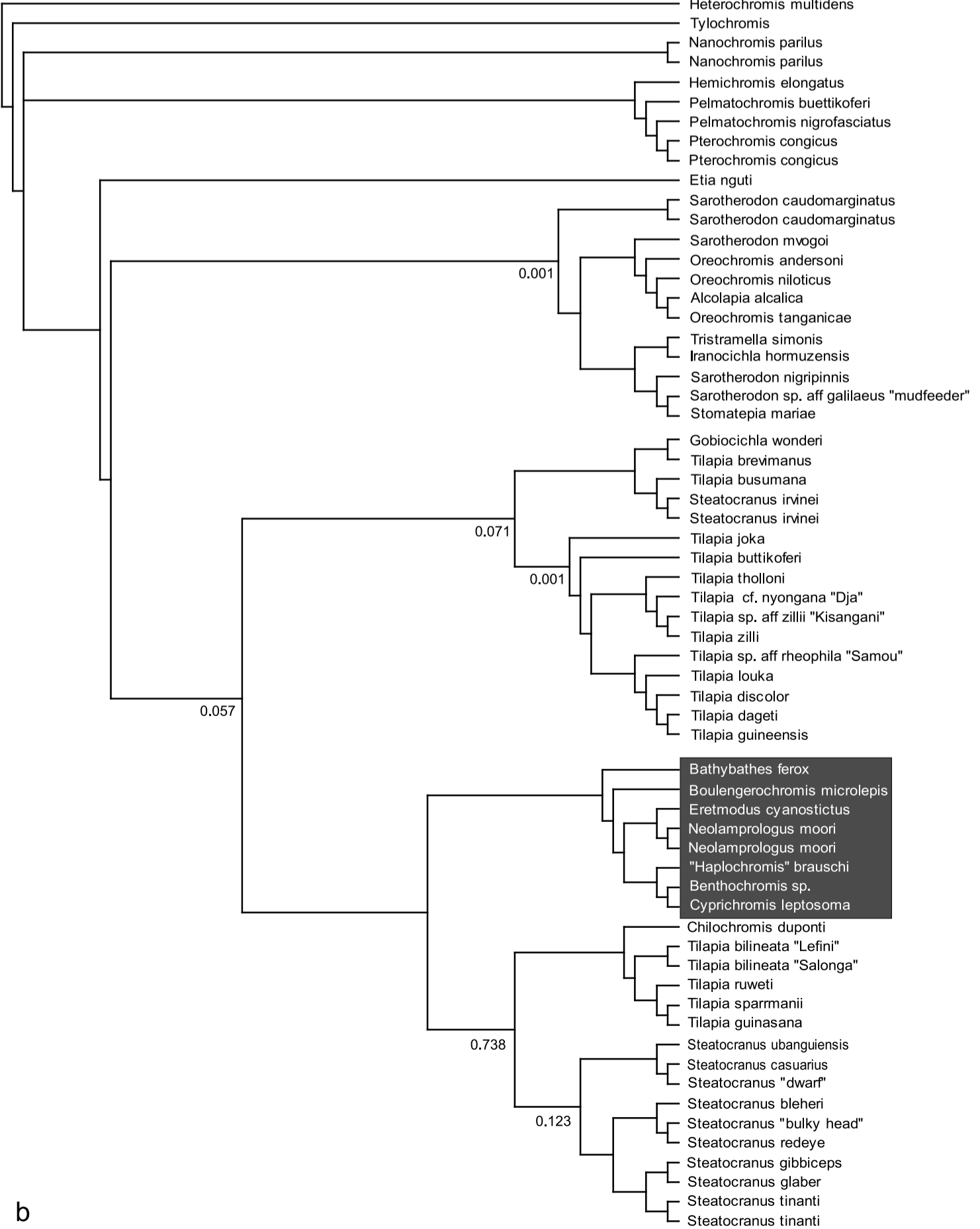

Supplement: Additional file 4 — Branch attachment frequencies in bootstrap replicates. Alternative positions of the single unstable taxon, T. mariae (a), and the EAR (b) in 1000 bootstrap topologies. The numbers, plotted on the ML tree, indicate fractions of bootstrap trees in which alternative branching patterns occur. [file 1471-2148-9-186-S4.pdf]

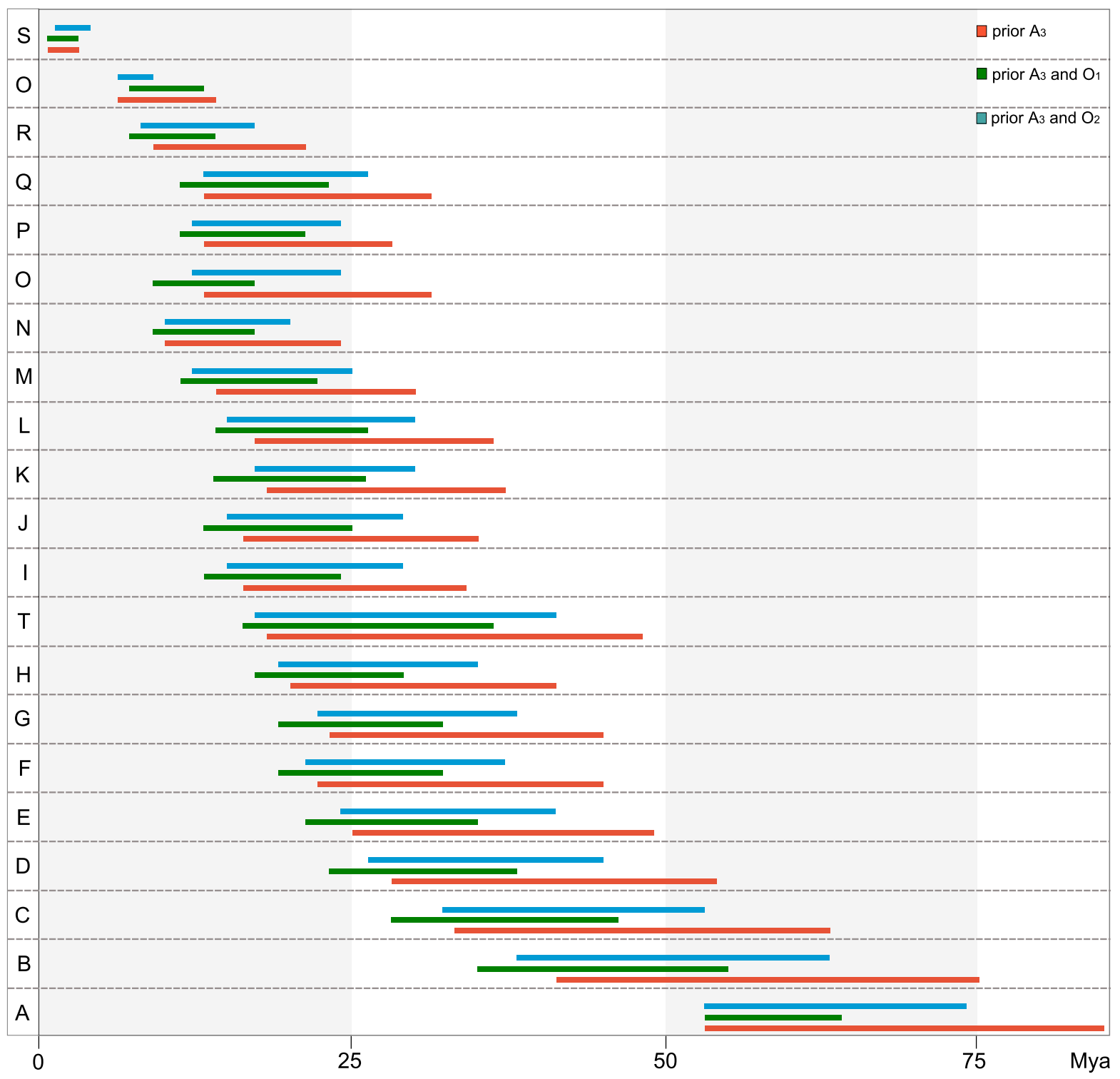

Supplement: Additional file 8 — Prior influence on divergence time estimates. The effects of different age constraints on the estimation of divergence times using BEAST. Bars indicate age ranges (95% credibility intervals) of different BEAST runs using either one single prior on the root (A3: 53–89 mya, based on published time intervals from [24]) or two priors, including the Oreochromis lorenzoi fossil (lower bound 5.98 mya) at two possible positions (O1 and O2) in the phylogeny (Figure 2). Using solely the root prior increases credibility intervals and renders the whole age estimation older. Inclusion of the fossil prior shifts intervals to a younger age. Large overlaps in estimates unite all three results and increase the plausibility of the presented results. Alternative positions of the Oreochromis lorenzoi† prior had no effect in age estimates using penalized likelihood (r8s). [file 1471-2148-9-186-S8.pdf]
