# Supplementary material for: The root of the East African cichlid radiations
Source: BMC Evol Biol. 2009 Aug 5;9:186. doi: 10.1186/1471-2148-9-186 (PMC2739198; doi:10.1186/1471-2148-9-186)
Supplement: Additional file 6 — Informal classification of African Cichlid fishes. Informal classification of African cichlid fishes. [file 1471-2148-9-186-S6.doc]

Informal classification of African cichlid genera with special reference to species level taxa previously referred to as “Tilapiini”

**Subfamily Heterochrominidinae** *Heterochromis*

**Subfamily Pseudocrenilabrinae**

**Tylochromini:** *Tylochromis*

**Hemichromini**: *Anomalochromis, Hemichromis*

**Pelmatochromini:** *Pelmatochromis, Pterochromis*

**Chromidotilapiini:** *Benitochromis*, *Chromidotilapia, Congochromis*, *Divandu*, *Limbochromis*, *Nanochromis*, *Parananochromis*, *Pelvicachromis*, *Teleogramma*, *Thysochromis*

**Haplotilapiini**

**Etiini:** *Etia nguti*

**Oreochromini:** *Alcolapia alcalica*, *Alcolapia* *grahami*, *Alcolapia latilabris,* *Alcolapia ndalani, Danakilia franchettii, Iranocichla hormuzensis, Konia eisentrauti, Konia dikume, Myaka myaka, Oreochromis amphimelas, Oreochromis andersonii, Oreochromis angolensis, Oreochromis aureus, Oreochromis chungruruensis, Oreochromis esculentus, Oreochromis hunteri, Oreochromis ismailiaensis, Oreochromis jipe, Oreochromis karomo, Oreochromis karongae, Oreochromis korogwe, Oreochromis lepidurus, Oreochromis leucostictus, Oreochromis lidole, Oreochromis macrochir, Oreochromis malagarsi, Oreochromis mortimeri, Oreochromis mossambicus, Oreochromis mweruensis, Oreochromis niloticus, Oreochromis pangani, Oreochromis placidus, Oreochromis rukwaensis, Oreochromis saka, Oreochromis salinicola, Oreochromis schwebischi, Oreochromis shiranus, Oreochromis spilurus, Oreochromis squamipinnis, Oreochromis tanganicae, Oreochromis upembae, Oreochromis urolepis, Oreochromis variabilis, Pungu maclareni, Sarotherodon steinbachi, Sarotherodon linnellii, Sarotherodon caroli, Sarotherodon lohbergeri, Sarotherodon caudomarginatus, Sarotherodon mvogoi, Sarotherodon occidentalis, Sarotherodon melanotheron, Sarotherodon nigripinnis, Sarotherodon galilaeus, Sarotherodon tournieri, Stomatepia pindu, Stomatepia mariae, Stomatepia mongo, Tristramella intermedia, Tristramella sacra, Tristramella simonis,*

**Austrotilapiini:**

**Clade AI (East African Radiation):**

**Boulengerochromini:** *Boulengerochromis microlepis*

**Hemibatini:** *Hemibates*

**Bathybatini:** *Bathybates*

**Trematocarini:** *Trematocara*

**Eretmodini**: *Eretmodus, Tanganicodus, Spathodus*

**Lamprologini:** *Altolamprologus*, *Chalinochromis*, *Julidochromis*, *Lamprologus*, *Lepidiolamprologus*, *Neolamprologus (incl. Variabilichromis)*, *Telmatochromis*

**Orthochromini:** *Orthochromis*

**Ectodini**: *Asprotilapia, Aulonocranus, Callochromis, Cardiopharynx, Cunningtonia, Cyathopharynx, Ectodus, Grammatotria, Lestradea, Microdontochromis, Opthalmotilapia*

**Cyprichromini**: *Cyprichromis*, *Paracyprichromis*

**Perissodini**: *Haplotaxodon*, *Perissodus (incl. Plecodus,Xenochromis)*

**Limnochromini**: *Baileychromis, Gnathochromis, Greenwoodochromis, Limnochromis, Reganochromis, Trematochromis benthicola*

**Benthochromini**: *Benthochromis*

**Cyphotilapiini**: *Cyphotilapia*

**Haplochromini**:

**Tropheini**: *“Ctenochromis” horei*, *«Gnathochromis» pfefferi, Limnotilapia, Lobochilotes, Petrochromis, Pseudosimochromis, Simochromis, Tropheus*

**Serranochromini:** *Chetia, Pharyngochromis, Sargochromis, Serranochromis, «Thoracochromis albolabris», «Thoracochromis» buysi*

**Lake Malawi clade:** all endemic Malawi genera

***Pseudocrenilabrus*-clade:** *Pseudocrenilabrus* (incl. *“Orthochromis” machadoi*)

***Ctenochromis* clade:** *Ctenochromis pectoralis*

***Astatoreochromis*** clade: *Astatoreochromis*

**Lake Victoria Superflock:** all «*Haplochromis*»

***“*Paraphyletic Rest”:** *Cyclopharynx*, “*Haplochromis” (incl. Astatotilapia, “Ctenochromis” polli*, *“Ctenochromis”oligacanthus*, *Rheohaplochormis*, *Thoracochromis*, *“Schwetzochromis”* *polyacanthus*, *“S.” stormsi*), *Schwetzochromis neodon*

**Clade AII**: *Chilochromis duponti, Tilapia baloni, Tilapia bilineata, T. guinasana, Tilapia sparrmanii, Tilapia ruweti*

**Clade AIII**: *Steatocranus* (except *“Steatocranus” irvinei*)

**Boreotilapiini**

**Clade BI:** *Gobiocichla ethewynnae, Gobiocichla wonderi*, *“Steatocranus”* *irvinei, Tilapia busumana*, *T. brevimanus*

**Clade BII:** *Tilapia. bakossiorum, Tilapia. bemini*, *Tilapia. buttikoferi*, *Tilapia. bythobates*, *Tilapia. cameronensis*, *Tilapia camerunensis*, *Tilapia cessiana*, *Tilapia coffea*, *Tilapia congica, Tilapia dageti, Tilapia deckerti, Tilapia discolor, Tilapia flava, Tilapia guineensis, Tilapia gutturosa, Tilapia imbriferna, Tilapia joka, Tilapia. kottae, Tilapia louka, Tilapia margaritacea, Tilapia nyongana, Tilapia rendalli, Tilapia spongotroktis, Tilapia tholloni, Tilapia thysi, Tilapia walteri, Tilapia zillii*

**Clade C**: *Tilapia cabrae*, *Tilapia mariae*

***Incertae sedis:*** *Tilapia rheophila*

Disclaimer: This list is not to be considered as published in the sense of the International Code of Zoological Nomenclature, and statements made herein are not made available for nomenclatural purposes from this document
