# Supplementary material for: The root of the East African cichlid radiations
Source: BMC Evol Biol. 2009 Aug 5;9:186. doi: 10.1186/1471-2148-9-186 (PMC2739198; doi:10.1186/1471-2148-9-186)
Supplement: Additional file 7 — Supplementary Information. Supplementary information for lab work (amplification, purification and sequencing of PCR products) and the choice of calibration priors for divergence time estimation. [file 1471-2148-9-186-S7.doc]

**Supplementary information**

The following primers were used for amplification and sequencing: partial mitochondrial 12S and 16S genes using primers L1091 and H1478 [1] and 16Sar-L and 16Sbr-H [2], the connecting part between the above mentioned fragments using primers fish12F1 and fish16SR1 as well as the internal primer fish 12SF2 for sequencing [3] and ND2 using primers ND2Met and ND2Trp [4]. Additionally, four nuclear protein coding genes, i.e. ENCI: primers ENCI\F85 and ENCI\R982, Ptr: primers Ptr\F458 and Ptr\R1248, Sh3px3: primers SH3PX3\_F461 and SH3PX3\_R1303 [5] and Tmo4c4 [6] and the first intron of the ribosomal protein coding gene S7, using primers S7RPEX1F50 and S7RPEX2R50 [7], were amplified and sequenced. Amplifications were performed in 10 µl volumes containing 5 µl Multiplex Mix (Qiagen), genomic DNA 1 µl, 0.8 µl of each Primer (2,5nmol), Q-Solution (Qiagen) and water. Amplifications of all fragments were carried out in 40 cycles according to the temperature profile: 15 min at 95 °C (initial denaturation), 30 s at 95 °C, 30 s at 55-60 °C, 60 - 90 s at 72 °C, and finally 10 min at 72 °C. PCR products were purified with ExoSAP-IT (USB) and diluted with 10 µl - 20 µl HPLC water, depending on product concentration. Sequencing was performed according to standard methods, using Big Dye 3.1. (Applied Biosystems). DNA sequences were read using an ABI 3130xl DNA sequencer (Applied Biosystems). Chromatograms were assembled using SeqMan v. 4.03 included in the Lasergene software package (DNASTAR) and proof read manually. Alignments were conducted using the Clustal W algorithm implemented in BioEdit v. 7.0.4.1 for coding genes and MUSLE v. 3.6 for non-coding genes and rRNA.

**Ambigous placement of *T. mariae***

Based on 1000 bootstrap and 2000 randomly chosen BI topologies branch attachment frequencies were calculated for the unstable taxon *Tilapia mariae* and the east African radiations using Phyutility v.2.2 [8]. Furthermore, statistical significance of likelihood differences between the best topology in which the EAR is nested within “Austrotilapiini” (Figure 2) and alternative topologies was tested using the approximately unbiased test (AU test) [9] implemented in the program Consel [10]. Eight topologies were tested, including all alternative topologies obtained via the branch attachment frequency test as well as topologies constructed solely based on the mitochondrial or nuclear datasets. The results are given in Table S3. A bootstrap homoplasy excess test was conducted. Bootstrap values for Autrotilapiini increased after excluding *T. mariae.* The effect was clearly higher for the focus taxon than for all other taxa iteratively excluded during the analysis (Figure 1).

**Choice of priors for the age estimation**

Using different approaches for dating cichlid divergence allow for a fairly exact placement within geological time periods, but can hardly provide precise values, due to a lack of adequate calibration points in the cichlid fossil record. Genner et al.[11] highlighted a bias of divergence estimates towards younger ages using cichlid fossils compared to geological time constraints based on Gondwana fragmentation. Age estimates based on cichlid fossils were half as young as those based on Gondwana calibrations [11]. However, constraining solely the root age might result in extremely high confidence intervals [12]. An alternative approach is to use younger geological time constraints, e.g. the lake ages, assuming that divergence of endemic clades took place after the formation of lake basins [e.g. 13-14]. These approaches resulted in heterogeneous age estimates for the origin of the EAR, ranging from 5 to > 35 mya [13,15]. Recently, however, molecular clock estimates based on non-cichlid teleost fossils resulted in plausible and tighter time intervals for basal cichlid nodes, which provide a novel source for calibration points in cichlids [16]. This study is based on these published time intervals, and only one cichlid fossil to calibrate a terminal node. *Oreochromis lorenzoi†* [17] was chosen for calibration as holo- and paratypes are in a well preserved state and a reliable species identification is possible, even in fossilized state.. Furthermore the phylogenetic placement within the African cichlid phylogeny is less ambiguous than for other fossils, as the “Oreochromini” are a clearly monophyletic group (Figure 2). This is clearly not the case for most other African cichlid fossils. For example, the oldest cichlid fossil known to date is *Mahengechromis* from Tanzania dated at about 46 mya [18,19]. Character states of key traits of this fossil are heterogeneous and a clear assignment to a cichlid tribe is not unambiguously possible [18,20]. Another cichlid fossil is a specimen described as cf. *Tylochromis?* (sic) from the Jebel Qatrani Formation, Fayum, in Egypt, dated at late Eocene/early Oligocene [21,22]. From this specimen only the pharyngeal jaw and teeth were preserved and the species determination was based on this. Here we follow a conservative approach using only one unambiguous fossil and test two alternative placements of this fossil at two slightly different nodes.

Reference List

1. Kocher TD, Thomas WK, Meyer A, Edwards SV, Paabo S, Villablanca FX, Wilson AC: Dynamics of mitochondrial-Dna evolution in animals - amplification and sequencing with conserved primers. *Proc. Natl. Acad. Sci. U.S.A.*1989, **86**:6196-6200.

2. Palumbi S, Martin A, Romano S: *The simple fool´s guide to PCR*. University of Hawaii Press, Honolulu, 1991.

3. Ruber L., Van Tassell JL, Zardoya R: Rapid speciation and ecological divergence in the American seven-spined gobies (Gobiidae, Gobiosomatini) inferred from a molecular phylogeny. *Evolution* 2003, **57**:1584-1598.

4. Kocher TD, Conroy JA, Mckaye KR, Stauffer JR, Lockwood SF: Evolution of NADH dehydrogenase subunit 2 in East African cichlid fish. *Mol. Phylogenet. Evol.* 1995, **4**:420-432.

5. Li CH, Orti G, Zhang G, Lu GQ: A practical approach to phylogenomics: the phylogeny of ray-finned fish (Actinopterygii) as a case study. *BMC Evol. Biol.* 2007, **7**:44.

6. Streelman JT, Zardoya R, Meyer A, Karl SA: Multilocus phylogeny of cichlid fishes (Pisces : Perciformes): Evolutionary comparison of microsatellite and single-copy nuclear loci*. Mol. Biol. Evol.* 1998, **15**:798-808.

7. Chow S, Hazama K: Universal PCR primers for S7 ribosomal protein gene introns in fish*. Mol. Ecol.* 1998, **7**:1255-1256.

8. Smith S A, Dunn CW: Phyutility: a phyloinformatics tool for trees, alignments and molecular data. *Bioinformatics* 2008, **24**:715-716.

9. Shimodaira H: An approximately unbiased test of phylogenetic tree selection. *Syst. Biol.* 2002, **51**:492-508.

10. Shimodaira H, Hasegawa M: CONSEL: for assessing the confidence of phylogenetic tree selection. *Bioinformatics* 2001, **17**:1246-1247.

11. Genner MJ, Seehausen O, Lunt DH, Joyce DA, Shaw PW, Carvalho GR, Turner GF: Age of cichlids: New dates for ancient lake fish radiations. *Mol. Biol. Evol.* 2007, **24**:1269-1282.

12. Renner SS, Zhang LB: Biogeography of the *Pistia* clade (Araceae): Based on chloroplast and mitochondrial DNA sequences and Bayesian divergence time inference. *Syst. Biol* 2004, **53**:422-432.

13. Salzburger W, Mack T, Verheyen E, Meyer A: Out of Tanganyika: Genesis, explosive speciation, key-innovations and phylogeography of the haplochromine cichlid fishes. *BMC Evol. Biol.* 2005, **5**:17.

14. Koblmüller S, Schliewen UK, Duftner N, Sefc K, Katongo C, Sturmbauer C: Age and spread of the haplochromine cichlid fishes in Africa. *Mol. Phylogenet. Evol.* 2008, **49**:153-169.

15. Koblmuller S, Sefc KM, Sturmbauer C: The Lake Tanganyika cichlid species assemblage: recent advances in molecular phylogenetics. *Hydrobiologia* 2008, **615**:5-20.

16. Azuma Y, Kumazawa Y, Miya M, Mabuchi K, Nishida M: Mitogenomic evaluation of the historical biogeography of cichlids toward reliable dating of teleostean divergences. *BMC Evol. Biol.* 2008, **8**:215.

17. Carnevale G, Sorbini C, Landini WT: *Oreochromis lorenzoi*, a new species of tilapiine cichlid from the late Miocene of central Italy. *Journal of Vertebrate Paleontology* 2003, **23**:508-516.

18. Murray AM: The oldest fossil cichlids (Teleostei : Perciformes): indication of a 45 million-year-old species flock. *Proc. R. Soc. Lond., B, Biol. Sci.* 2001, **268**:679-684.

19. Murray AM: Eocene cichlid fishes from Tanzania, East Africa. *Journal of Vertebrate Paleontology* 2000, **20**:651-664.

20. Murray AM: The fossil record and biogeography of the Cichlidae (Actinopterygii : Labroidei). *Biol. J. Linn. Soc. Lond.* 2001, **74**:517-532.

21. Murray AM: Late Eocene and early Oligocene teleost and associated ichthyofauna of the Jebel Qatrani Formation, Fayum, Egypt. *Palaeontology* 2004, **47**: 711-724.

22. Murray AM: Lower pharyngeal jaw of a cichlid fish (Actinopterygii; Labroidei) from an early Oligocene site in the Fayum, Egypt. *Journal of Vertebrate Paleontology* 2002, **22**: 453-455.

**Figures**

**Figure S1**

Boxplot showing the distribution of bootstrap support values (%) for the “Austrotilapiini”. Each specimen was removed iteratively from the dataset (resulting in N= 63 experiments) and 1000 bootstrap replicates were calculated using ML. Outliers are shown as asterisks. Bootstrap support values clearly increased (from initially 56 to 86) after exclusion of *T. mariae*. Removal of all other taxa did not cause this effect.

**Figure S2**

Maximum likelihood phylogeny for dataset B based on 992bp of ND2. Sequences are taken from GenBank (N=263) and taxa added from our dataset A (N=38). Focus clades are marked with black bars and BS support values are given only for those clades. All focus clades (well supported clades from dataset A) were recovered as monophyletic in this tree, despite lower data density and higher taxon sampling. One *Tilapia discolor* sequence from a specimen of the Pra River (Ghana) taken from GenBank ^24^ is nested within *T. busumana* in clade BI instead of being sister to our conspecific and positively identified *T. discolor* of its type locality at Lake Bosumtwi Ghana. As no specimen vouchers of this specimen are available, we assume that either misidentification or mitochondrial introgression of sympatric *T. busumana* is the reason for this discrepancy.

**Figure S3**

Alternative positions of the single unstable taxon, *T. mariae* (a)*,* and the EAR (b) in 1000 bootstrap topologies. The numbers, plotted on the ML tree, indicate fractions of bootstrap trees in which alternative branching patterns occur.

**Figure S4**

Time divergence estimates for African cichlids. The chronogram was calculated based on the BI consensus tree. Divergence times were estimated using Bayesian analysis implemented in BEAST. The following time constraints were used: **A_1_** 53-84 mya (uniform prior), published age estimate based on non-cichlid fossils [17] and **O_1_** 5.98 mya (lower bound), the age estimate for *Oreochromis lorenzoi†* [18]. The chronogram shows 95% credibility intervals (HPC, grey bars). For nodes marked with letters age estimates (95% HPC and mean heights) were calculated. Calibration points (**O_1_** and **A_1_**) are marked with black squares. For simplification clear monophyletic groups were combined (shown as triangles).

**Figure S5**

The effects of different age constraints on the estimation of divergence times using BEAST. Bars indicate age ranges (95% credibility intervals) of different BEAST runs using either one single prior on the root (A_3_: 53-89 Mya, based on published time intervals from ^17^) or two priors, including the *Oreochromis lorenzoi†* fossil (lower bound 5.98 Mya) at two possible positions (O_1_ and O_2_) in the phylogeny (Figure 2). Using solely the root prior increases credibility intervals and renders the whole age estimation older. Inclusion of the fossil prior shifts intervals to a younger age. Large overlaps in estimates unite all three results and increase the plausibility of the presented results. Alternative positions of the *Oreochromis lorenzoi†* prior had no effect in age estimates using penalized likelihood (R8s).
